# Executive, language and fluency dysfunction are markers of localised TDP-43 cerebral pathology in non-demented ALS

**DOI:** 10.1136/jnnp-2019-320807

**Published:** 2019-09-12

**Authors:** Jenna M Gregory, Karina McDade, Thomas H Bak, Suvankar Pal, Siddharthan Chandran, Colin Smith, Sharon Abrahams

**Affiliations:** 1 Centre for Clinical Brain Sciences, University of Edinburgh, Edinburgh, UK; 2 Euan MacDonald Centre for Motor Neurone Disease Research, University of Edinburgh, Edinburgh, UK; 3 School of Philosophy, Psychology and Language Science, University of Edinburgh, Edinburgh, UK

**Keywords:** ECAS, TDP-43, cognition, ALS, Neuropathology

## Abstract

**Objective:**

Approximately 35% of patients with amyotrophic lateral sclerosis (ALS) exhibit mild cognitive deficits in executive functions, language and fluency, without dementia. The precise pathology of these extramotor symptoms has remained unknown. This study aimed to determine the pathological correlate of cognitive impairment in patients with non-demented ALS.

**Methods:**

In-depth neuropathological analysis of 27 patients with non-demented ALS who had undergone cognitive testing (Edinburgh Cognitive and Behaviour ALS Screen (ECAS)) during life. Analysis involved assessing 43 kDa Tar-DNA binding protein (TDP-43) accumulation in brain regions specifically involved in executive functions, language functions and verbal fluency to ascertain whether functional deficits would relate to a specific regional distribution of pathology.

**Results:**

All patients with cognitive impairment had TDP-43 pathology in extramotor brain regions (positive predictive value of 100%). The ECAS also predicted TDP-43 pathology with 100% specificity in brain regions associated with executive, language and fluency domains. We also detected a subgroup with no cognitive dysfunction, despite having substantial TDP-43 pathology, so called mismatch cases.

**Conclusions:**

Cognitive impairment as detected by the ECAS is a valid predictor of TDP-43 pathology in non-demented ALS. The profile of mild cognitive deficits specifically predicts regional cerebral involvement. These findings highlight the utility of the ECAS in accurately assessing the pathological burden of disease.

## Introduction

Amyotrophic lateral sclerosis (ALS) has a heterogeneous clinical manifestation and is accompanied by cognitive and/or behaviour dysfunction in 35% of patients, with an additional 15% exhibiting a frontotemporal dementia (FTD) typically a behavioural variant.^1–3^ Patients show executive dysfunction, social cognition, language and letter fluency deficits, in addition to abnormal behaviour, notably apathy.^1^ These extramotor symptoms are associated with reduced survival. However, their precise pathology has remained unknown.

The presence of 43 kDa Tar-DNA binding protein (TDP-43), whose pathological misfolding and accumulation is observed in the brains and spinal cord of patients with ALS, except for cases of superoxide dismutase 1 *(SOD1*) mutation, suggests a common underlying process.^4^ TDP-43 is a predominantly nuclear protein involved in transcriptional regulation. In ALS, TDP-43 is pathologically phosphorylated and C-terminally truncated in cytoplasmic aggregates.^5^ The toxicity may be partly loss and partly gain-of-function: insoluble aggregates inhibit TDP-43 function in transcriptional regulation, while cytoplasmic aggregates can lead to the dysregulation of proteostasis and sequester other aggregation prone proteins, causing further cytotoxicity and contributing to cell death.^6 7^

In ALS brains, TDP-43 pathology can be widespread in cases of ALS-related/FTD and some have suggested a sequential spread.^8^ A non-specific association was found between the presence of cognitive impairment (yes/no) as measured by a dementia screening tool, the Mini Mental State Examination and moderate to severe TDP-43 pathology.^9^ Using more detailed neuropsychology, Prudlo and colleagues divided the patients with ALS into three clinical groups: ALS with no cognitive impairment, ALS with cognitive impairment and ALS-FTD. A significant difference was demonstrated in the severity of pathologically misfolded TDP-43 inclusions, between patients with ALS-FTD and the combined non-demented clinical groups (ALS with or without cognitive impairment); however, there was no difference between the two non-demented groups: ALS with cognitive impairment and ALS with no cognitive impairment.^10^ An association between the cognitive profile in patients with non-demented ALS with cognitive impairment and TDP-43 has not been shown previously. Furthermore, the association between specific cognitive functions (executive, language and fluency) tested during life using the same cognitive tool and the distribution of postmortem TDP-43 pathology over several distinct brain regions has never been demonstrated. Studies are limited as cohorts have been assessed by different cognitive tests not designed for ALS or physical disability. The introduction of, the Edinburgh Cognitive and Behavioural ALS Screen (ECAS) enables a more cohesive approach. This standardised tool assesses ALS-related cognitive deficits (verbal fluency, executive and language functions) and is sensitive to milder cognitive impairments.^1 11^ Using the ECAS, we have been able to demonstrate an association between the presence of cognitive impairment and synaptic loss in the prefrontal cortex of patients with ALS.^7^ Here we are the first to examine the relationship between the profile of cognitive impairment as detected by the ECAS in non-demented ALS and the localised distribution of TDP-43.

The aim was to determine the pathological correlate of cognitive impairment in patients with non-demented ALS. Our hypotheses are that (1) the specific profile of cognitive impairment in patients with non-demented ALS (executive, fluency and language dysfunction) is associated with TDP-43 pathology in corresponding brain regions identified a priori and (2) the ECAS assessed in vivo is a good predictor of TDP-43 pathology postmortem.

## Methods

### Case identification

We identified, from the Medical Research Council (MRC) Edinburgh Brain Bank, 27 ALS cases who had undergone neuropsychological testing with the ECAS during life. Additionally, all cases had separately undergone whole genome sequencing. All clinical data including the ECAS were collected as part of Scottish Motor Neuron Disease Register (SMNDR) and Care Audit Research and Evaluation for Motor Neuron Disease (CARE-MND) platform and all patients consented to the use of their data during life. All postmortem tissue was collected via the Edinburgh Brain Bank in line with the Human Tissue (Scotland) Act.

### Cognitive assessment

Cognitive impairment was measured using the ECAS, and all assessments were performed according to the standard procedure (https://ecas.psy.ed.ac.uk/). The ECAS is specifically designed to assess the profile of cognitive impairment in ALS and is adapted for patients with physical disability. It consists of 16 subtests across five cognitive domains: executive functions, language functions and letter fluency which combine to produce and ALS-specific score, while memory and visuospatial functions combine to produce an ALS-non-specific score. The ECAS also includes a carer behavioural interview based on criteria for diagnosing behavioural variant FTD.^2^

The ECAS provides high sensitivity and specificity in the detection of mild cognitive deficits in ALS against gold standard neuropsychological assessment using published cut-off scores for abnormality; has been validated extensively in many different diverse populations of patients with ALS and against other neuropsychological screening tools and batteries.^3 12–19^

The final ECAS score (maximum score of 136) is made up of the ALS-specific (maximum score of 100) and ALS-non-specific (maximum score of 36) scores. A person can be judged to be cognitively impaired (ALSci) if they have failed the ECAS (see supplemental information for diagnosing ALS-frontotemporal spectrum disorder using the ECAS). Here we used published cut-offs for the ECAS total and ALS-specific scores which have shown maximum sensitivity and specificity to detect ALS-related cognitive impairment against extensive neuropsychological assessment.^12^ The cut-offs for abnormality are ECAS Total 105/136 or less, ALS-specific score 77/100 or less. Abnormality cut-off scores for the cognitive subdomains are 33/48 for executive function, 14/24 for fluency and 26/28 for language function.^3^ ALS with behavioural impairment (ALSbi) was defined as the presence of apathy or two other behaviour features as defined by the consensus.^2^

10.1136/jnnp-2019-320807.supp1 Supplementary data

### Histology and neuropathological assessment

Brain tissue was taken at postmortem from standardised Brodmann areas (BA) and fixed in 10% formalin for a minimum of 24 hours. Tissue was dehydrated in an ascending alcohol series (70%–100%) followed by three successive 4-hour washes in xylene. Three successive 5-hour paraffin wax embedding stages were performed followed by cooling and sectioning of the formalin-fixed paraffin embedded (FFPE) tissue on a Leica microtome in 4 µm sections on to a superfrost microscope slide. Sections were dried overnight at 40°C and immunostaining was performed using the Novolink Polymer detection system with the Proteintech anti-phospho(409-410)-TDP-43 antibody at a 1 in 1000 dilution and 3,3′-diaminobenzidine chromogen and counterstained with haematoxylin, according to standard operating procedures.

**Figure 1:**
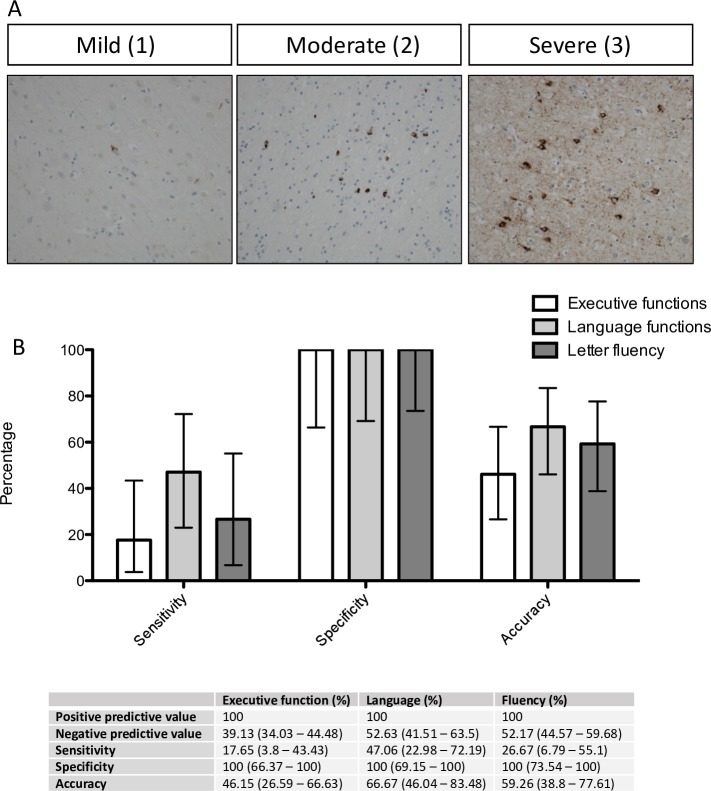
Subdomain cognitive dysfunction detected by the ECAS relates to specific regional distribution of TDP-43 pathology. (A) Pathological TDP-43 staining, demonstrating characteristic cytoplasmic aggregation and nuclear clearance of TDP-43. Images are taken at 20× magnification, illustrating mild, moderate and severe scoring of pathology. (B) Sensitivity and specificity analysis assessing the utility of ECAS subdomains in predicting TDP-43 pathology in corresponding brain regions, demonstrating high positive predictive value. Values are percentage with 95% CIs. ECAS, Edinburgh Cognitive and Behavioural Amytrophic lateral sclerosis Screen.

For the purpose of later analyses, regions of the brain were grouped according to their association with (1) executive, (2) language functions or (3) letter fluency based on functional imaging and pathological studies.^20–22^ Brain regions which have been previously associated with executive functions included the orbitofrontal cortex (BA11/12), ventral anterior cingulate (BA24), dorsolateral prefrontal cortex (BA46 and BA9) and the medial prefrontal cortex (BA6). Regions associated with language functions included the inferior frontal gyrus (Broca’s area; BA44/45), transverse temporal area (Heschl’s gyri; BA41/42), middle and inferior temporal gyri (BA20/21) and the angular gyrus (BA39). In accordance with the underlying cognitive processes involved in fluency regions associated with fluency overlap between those of executive and language functions including the prefrontal cortex (BA9), inferior frontal gyrus (Broca’s area; BA44/45), ventral anterior cingulate (BA24) and the transverse temporal area (Heschl’s gyri; BA41/42). For a summary of these regions, see table 1. TDP-43 pathology was graded by two independent pathologists (pretest reviewer concordance acceptability was set at >0.66; therefore, disagreements were solved by discussion, resulting in 100% concordance), using a semiquantitative scoring system from 0 to 3: 0=no TDP-43 pathology; 1=mild (up to five affected cells in at least one 20× field of view per section); 2=moderate (5–15 affected cells in at least one 20× field of view per section); 3=severe (>15 cells affected in at least one 20× field of view per section) (figure 1A). Cases defined as having no pathology had a score of 0, while those with pathology had a score from 1 to 3 for each region. This scoring was applied to neuronal and glial cell population (based on established neuropathological knowledge of cell morphology using haematoxylin counterstain). Neuronal cells were determined by their larger cell size (nucleus surrounded by a large volume of cytoplasm, the presence of dendrites and cortical layer location). Glia were designated based on small cell size with oval to round nuclei with compact chromatin, small rim of cytoplasm. Assessors were blinded to all demographic and clinical information. Regions grouped by their functional significance had to have three out of four (or two out of three for fluency) regions with TDP-43 pathology present to be classified as positive for pathology and the pathology had to be at least one in these regions.

**Table 1:** Functional and clinical correlates of chosen brain regions identified a priori for analysis

Brodmann area	Functional correlate	Clinical correlate
BA6, BA11, BA24, BA46	Executive	ECAS (ALS specific subtests and scores)
BA9, BA44, BA41	Fluency	
BA20, BA39	Language	
BA19	Visuospatial	ECAS (ALS non-specific subtests and scores)
Antihippocampus Post-hippocampus Amygdala	Memory	

ALS, amyotrophic lateral sclerosis; ECAS, Edinburgh Cognitive and Behavioural ALS Screen.

### Statistical methods

**Figure 2:**
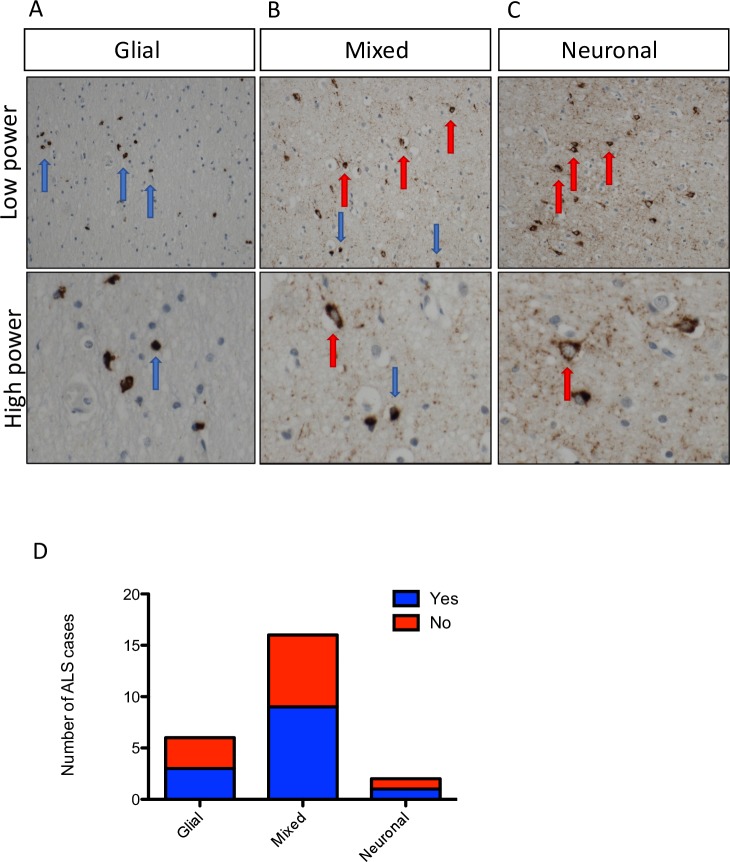
Cell-type specific TDP-43 pathology differs between individuals. Pathological TDP-43 staining, demonstrating (A) predominantly glial inclusions; (B) mixed glial and neuronal inclusions and (C) predominantly neuronal inclusions. Images are taken at 20× magnification; red arrows indicate neurons and blue arrows indicate glial cells. (D) Frequency distribution demonstrating the number of cases with each of these characteristic cell-type specific inclusions. Each column is subdivided into red (evidence of cognitive dysfunction) and blue (no evidence of cognitive dysfunction) showing that there is no apparent link between cell-type specific TDP-43 accumulation and cognitive dysfunction in the cases that we analysed. ALS, amyotrophic lateral sclerosis; TDP-43, 43 kDa Tar-DNA binding protein.

Sensitivity, specificity, positive and negative predictive values are expressed as percentages. CIs for sensitivity and specificity are exact Clopper-Pearson CIs. CIs for the likelihood ratios are calculated using the Log method. CIs for the predictive values are the standard logit CIs. GraphPad Prism V.5.0 was used to present graphical data. Non-parametric analysis of (1) median survival was performed using Mann-Whitney U test and (2) time between ECAS testing and death was performed using two-tailed Fisher’s exact text. Shapiro-Wilk test was performed to ensure data were normally distributed. One-way analysis of variance (ANOVA) was used to compare cognitively impaired and unimpaired cases with different cell-type specific distributions of TDP-43 (figure 2D).

## Results

### Cohort demographics

Twenty-seven patients with ALS (clinically established by El Escorial criteria) from the Edinburgh Brain Bank who had undergone neuropsychological testing during life using the ECAS were identified (table 2). No patient had a diagnosis of dementia in their clinical notes. All the patients had undergone whole genome sequencing identifying nine individuals with a confirmed genetic ALS-associated mutation, with five *C9orf72* cases, two cases carrying the *SOD1* (I114T) Scottish founder mutation and two cases with mutations of uncertain significance in the *NEK1* gene.^23^ Seventy-two per cent of cases identified had limb onset disease, 23% had bulbar onset disease and one individual had a combined limb and bulbar onset of disease. Seven individuals (26% of the cohort) were classified as having ALSci (see table 3). Behavioural data was available on nine cases of which three were classified as ALSbi, one of which carried the I114T *SOD1* mutation. No individual showed ALScibi, likely reflecting the small number of individuals with available behavioural data (nine individuals within the cohort). Eleven individuals demonstrated cognitive impairment in specific subdomains; executive function only (one individual), language (six individuals) and fluency (two individuals) with two individuals having a mixed deficit affecting all three domains (table 3). Only one case with language dysfunction was also impaired on the memory subtest, but none were impaired on visuospatial testing.

**Table 2:** ECAS cohort demographics

Demographics	n (%)	Median (months)	IQR (months)
Age			
At disease onset		56	48–60.75
At death		63	50.25–66
Disease duration			31.5–98.75
Sex			
Male	12 (45)		
Female	15 (55)		
Disease onset			
Upper limb	8 (36)		
Lower limb	8 (36)		
Bulbar	5 (23)		
Combined	1 (5)		
Genetic diagnosis			
*C9orf72*	6 (23)		
*SOD1 (I114T)*	2 (9)		
*NEK1*	2 (9)		
No known mutation (all had WGS)	17 (59)		
Cognitive impairment (ECAS)	11 (40)		
Executive dysfunction	1 (4)		
Language	6 (22)		
Fluency	2 (7)		
Combined	2 (7)		

Summary of clinical findings and demographics of patient cohort.

ECAS, Edinburgh Cognitive and Behavioural Amyotrophic lateral sclerosis Screen; WGS, whole-genome sequencing.

**Table 3:** Cognitive scoring of ALS cohort

**Behavioural impairment**		**Cognitive assessment**								
**ID**	Overall ALSci (Y/N)	**ALS Specific**	**ALS non-specific**	**ECAS total**	**Executive**	**Language**	**Fluency**	**Memory**	**Visuospatial**	**Behaviour**
**Case 1**	N	92	33	125	42	28	22	22	11	4
**Case 2 (*NEK1***)	N	86	30	116	38	28	20	18	12	2
**Case 3 (*SOD1***)	N	87	31	118	41	28	18	19	12	2
Executive dysfunction		**Cognitive assessment**								
**ID**	**Overall ALSci (Y/N**)	**ALS specific**	**ALS non-specifc**	**ECAS total**	**Executive**	**Language**	**Fluency**	**Memory**	**Visuospatial**	**Behaviour**
**Case 4 (*C9orf72***)	Y	76	26	102	31	27	18	14	12	ND
Language dysfunction		**Cognitive assessment**								
**ID**	**Overall ALSci (Y/N**)	**ALS specific**	**ALS non-specifc**	**ECAS total**	**Executive**	**Language**	**Fluency**	**Memory**	**Visuospatial**	**Behaviour**
**Case 5 (*C9orf72***)	N	85	27	112	39	26	20	15	12	0
**Case 6 (*C9orf72***)	Y	76	26	102	35	23	18	14	12	ND
**Case 7**	Y	81	20	101	37	26	18	8	12	ND
**Case 8 (*C9orf72***)	Y	74	26	100	36	20	18	14	12	ND
**Case 9**	N	88	26	114	44	26	18	14	12	ND
**Case 10**	N	85	26	111	36	25	24	14	12	0
Fluency dysfunction		**Cognitive assessment**								
**ID**	**Overall ALSci (Y/N**)	**ALS specific**	**ALS non-specifc**	**ECAS total**	**Executive**	**Language**	**Fluency**	**Memory**	**Visuospatial**	**Behaviour**
**Case 11**	Y	75	30	105	39	28	8	18	12	ND
**Case 12**	N	82	33	115	44	28	10	21	12	ND
Combined deficit: executive, language and fluency		**Cognitive assessment**								
**ID**	**Overall ALSci (Y/N**)	**ALS specific**	**ALS non-specifc**	**ECAS total**	**Executive**	**Language**	**Fluency**	**Memory**	**Visuospatial**	**Behaviour**
**Case 13**	Y	65	26	91	32	25	8	15	11	0
**Case 14**	Y	62	28	90	32	22	8	16	12	ND
Unimpaired		**Cognitive assessment**								
**ID**	**Overall ALSci (Y/N**)	**ALS specific**	**ALS non-specifc**	**ECAS total**	**Executive**	**Language**	**Fluency**	**Memory**	**Visuospatial**	**Behaviour**
**Case 15 (*C9orf72***)	N	83	31	114	39	28	16	21	10	0
**Case 16**	N	87	33	120	41	28	18	21	12	ND
**Case 17**	N	97	35	132	47	28	22	23	12	ND
**Case 18**	N	93	32	125	45	28	20	20	12	1
**Case 19 (*NEK1***)	N	88	30	118	42	28	18	18	12	ND
**Case 20**	N	85	29	114	39	28	18	17	12	ND
**Case 21**	N	86	30	116	40	28	18	18	12	ND
**Case 22**	N	82	33	115	35	27	20	21	12	0
**Case 23(*C9orf72***)	N	86	32	118	40	28	18	20	12	0
**Case 24**	N	83	29	112	38	27	18	17	12	ND
**Case 25**	N	82	29	111	36	28	18	17	12	ND
**Case 26(*SOD1***)	N	82	28	110	34	28	20	16	12	ND
**Case 27**	N	92	32	124	44	28	20	20	12	ND

Summary of ECAS scores for each patient in the cohort, subdivided by subdomain deficits.

ALS, amyotrophic lateral sclerosis; ALSci, cognitively impaired ALS; ECAS, Edinburgh Cognitive and Behavioural ALS Screen; ND, not done.

### Cognitive dysfunction detected by the ECAS is associated with pathological TDP-43 accumulation

To determine whether cognitive impairment as detected by the ECAS was a good predictor of extramotor pathology in ALS we first assessed whether patients with impaired ECAS scores exhibited extramotor pathology assessed by TDP-43 aggregation in postmortem tissue.

Three out of 27 patients met criteria for ALSbi; one of these had a NEK1 mutation, one had a SOD1 mutation and one was a sporadic case. The brain areas thought to predominantly be associated with behavioural dysfunction are (1) the orbitofrontal cortex (BA11/12), (2) ventral anterior cingulate (BA24) and (3) medial prefrontal cortex (BA6). There was no TDP-43 present in the case with SOD1 mutation, in keeping with previous literature; however, the remaining two cases did have TDP-43 inclusions in two or more of these brain regions (table 4). 7 out of our cohort of 27 patients met criteria for ALSci. All 7 of these patients exhibited TDP-43 pathology in extramotor brain regions (table 4). Therefore, in every case exhibiting ALSci, there was evidence of extramotor TDP-43 pathology. There were six false negatives, whereby TDP-43 pathology in extramotor brain regions was not accompanied by an impairment in the ECAS.^24^ These data taken together were used to assess the diagnostic accuracy of ECAS in predicting TDP-43 pathology in extramotor brain regions resulting in a diagnostic accuracy of 66.67% (95% CI 46.04 to 83.48), with a sensitivity of 43.75% (95% CI 19.75 to 70.12) and a specificity of 100% (95% CI 71.51 to 100).

**Table 4:** Postmortem pathological scoring

Behavioural impairment		**Postmortem assessment of TDP-43 accumulation**														
		**Cell type**	**Motor**	**Executive function**					**Language**				**Sensory**	**Other**		
**ID**	**Disease duration**		**BA4**	**BA6**	**BA11**	**BA24**	**BA46**	**BA9**	**BA44**	**BA41**	**BA20**	**BA39**	**BA19**	**Ant hip**	**Post hip**	**Amygdala**
**Case 1**	24	Neuronal	1	1	0	1	0	0	1	1	1	1	1	1	1	1
		Glial	1	1	0	1	1	0	1	1	1	1	1	1	1	1
**Case 2 *(NEK1***)	159	Neuronal	3	3	1	2	2	2	2	2	2	1	2	3	3	3
		Glial	3	2	1	1	2	2	2	1	1	1	1	2	2	2
**Case 3 (*SOD1***)	98	Neuronal	0	0	0	0	0	0	0	0	0	0	0	0	0	0
		Glial	0	0	0	0	0	0	0	0	0	0	0	0	0	0
Executive dysfunction		**Postmortem assessment of TDP-43 accumulation**														
		**Cell type**	**Motor**	**Executive function**					**Language**				**Sensory**	**Other**		
**ID**	**Disease duration**		**BA4**	**BA6**	**BA11**	**BA24**	**BA46**	**BA9**	**BA44**	**BA41**	**BA20**	**BA39**	**BA19**	**Ant hip**	**Post hip**	**Amygdala**
**Case 4 (*C9orf72***)	97	Neuronal	2	1	1	2	1	0	1	1	0	1	0	1	1	1
		Glial	0	0	0	0	0	0	0	0	0	0	0	0	0	0
Language dysfunction		**Postmortem assessment of TDP-43 accumulation**														
		**Cell type**	**Motor**	**Executive function**					**Language**				**Sensory**	**Other**		
**ID**	**Disease duration**		**BA4**	**BA6**	**BA11**	**BA24**	**BA46**	**BA9**	**BA44**	**BA41**	**BA20**	**BA39**	**BA19**	**Ant hip**	**Post hip**	**Amygdala**
**Case 5 (*C9orf72***)	58	Neuronal	2	2	1	1	1	1	1	1	1		1	0	1	1
		Glial	3	2	1	1	1	1	1	1	1		1	0	0	1
**Case 6 (*C9orf72***)	58	Neuronal	1	1	1	1	1	0	0	1	0	0	0	0	0	0
		Glial	2	1	0	1	0	0	0	1	0	0	0	0	0	0
**Case 7**	17	Neuronal	3	0	1	0	1	1	1	1	1	1	0	0	0	0
		Glial	3	1	1	1	1	1	1	0	0	1	0	0	0	0
**Case 8 (*C9orf72***)	60	Neuronal	3	1	1	1	1	1	1	1	1	1	0	0	0	0
		Glial	3	1	1	1	1	1	1	1	1	1	0	0	0	0
**Case 9**	150	Neuronal	2	1	0	1	0	1	1	1	1	1	0	0	0	0
		Glial	2	1	0	1	1	1	1	1	1	1	0	0	0	0
**Case 10**	13	Neuronal	3	2	1	2	1	1	1	1	1	1	0	0	0	0
		Glial	3	2	1	2	1	1	1	1	1	1	0	0	0	0
Fluency dysfunction		**Postmortem assessment of TDP-43 accumulation**														
		**Cell type**	**Motor**	**Executive function**					**Language**				**Sensory**	**Other**		
**ID**	**Disease duration**		**BA4**	**BA6**	**BA11**	**BA24**	**BA46**	**BA9**	**BA44**	**BA41**	**BA20**	**BA39**	**BA19**	**Ant hip**	**Post hip**	**Amygdala**
**Case 11**	14	Neuronal	2	3	3		3	3	3	2	2	2	2	2	3	3
		Glial	2	3	2		3	2	2	1	2	2	2	2	2	2
**Case 12**	99	Neuronal	0	1	0	0	0	1	0	0	0	1	0	0	0	0
		Glial	2	2	0	1	1	1	1	1	1	2	1	0	0	0
Combined deficit: executive, language and fluency		**Postmortem assessment of TDP-43 accumulation**														
		**Cell type**	**Motor**	**Executive function**					**Language**				**Sensory**	**Other**		
**ID**	**Disease duration**		**BA4**	**BA6**	**BA11**	**BA24**	**BA46**	**BA9**	**BA44**	**BA41**	**BA20**	**BA39**	**BA19**	**Ant hip**	**Post hip**	**Amygdala**
**Case 13**	20	Neuronal	1	2	2	2	1	1	1	1	2	1	1	2	2	2
		Glial	1	2	2	2	2	2	1	1	2	1	1	2	2	1
**Case 14**	130	Neuronal	1	1	1	1	1	0	1	1	2	1	0	2	1	1
		Glial	1	1	1	1	1	0	1	1	2	1	0	2	1	1
Unimpaired		**Postmortem assessment of TDP-43 accumulation**														
		**Cell type**	**Motor**	**Executive function**					**Language**				**Sensory**	**Other**		
**ID**	**Disease duration**		**BA4**	**BA6**	**BA11**	**BA24**	**BA46**	**BA9**	**BA44**	**BA41**	**BA20**	**BA39**	**BA19**	**Ant hip**	**Post hip**	**Amygdala**
**Case 15 (*C9orf72***)	109	Neuronal	1	1	1	1	1	1	1	1	1	1	1	1	1	1
		Glial	2	2	1	1	2	2	1	1	1	1	1	1	1	1
**Case 16**	134	Neuronal	1	2	1	1	2	1	2	1	1	1	1	2	2	2
		Glial	1	2	1	1	2	1	2	1	1	1	1	2	2	2
**Case 17**	30	Neuronal	0	0	0	0	0	0	0	0	0	0	0	0	0	0
		Glial	0	0	0	0	0	0	0	0	0	0	0	0	0	0
**Case 18**	16	Neuronal	1	0	0	0	0	0	0	1	0	1	1	0	0	1
		Glial	2	0	0	0	0	0	0	1	0	2	0	0	0	1
**Case 19 (*NEK1***)	119	Neuronal	1	0	0	0	0	0	0	0	0	0	0	0	0	0
		Glial	0	0	0	0	0	0	0	0	0	0	0	0	0	0
**Case 20**	54	Neuronal	1	0	0	0	0	0	0	0	0	0	0	0	0	0
		Glial	1	0	0	0	0	0	0	0	0	0	0	1	0	0
**Case 21**	46	Neuronal	1	0	0	0	0	0	0	0	0	0	0	0	0	0
		Glial	2	0	0	0	0	0	0	1	0	0	0	0	0	0
**Case 22**	31	Neuronal	0	1	0	0	1	0	0	0	0	0	0	0	0	0
		Glial	0	1	0	0	1	0	0	0	0	0	0	0	0	1
**Case 23 (*C9orf72***)	33	Neuronal	2	2	1	2	1	1	1	1	1	1	1	1	1	1
		Glial	2	2	0	2	1	0	1	1	1	1	1	1	1	1
**Case 24**	64	Neuronal	1	0	0	1	0	0	0	0	0	0	0	0	0	0
		Glial	1	0	0	1	0	0	0	0	0	0	0	0	0	0
**Case 25**	45	Neuronal	1	0	0	0	0	0	0	0	0	0	0	0	0	0
		Glial	1	0	0	0	0	0	0	0	0	0	0	0	0	0
**Case 26 (*SOD1***)	67	Neuronal	0	0	0	0	0	0	0	0	0	0	0	0	0	0
		Glial	0	0	0	0	0	0	0	0	0	0	0	0	0	0
**Case 27**	16	Neuronal	0	1	1	1	1	1	0	0	0	0	0	0	0	0
		Glial	0	1	1	1	1	1	0	0	0	0	0	0	0	0

Summary of post-mortem TDP-43 pathology for each patient in the cohort, subdivided by ECAS subdomain deficits. Brain regions are grouped by function into: (1) motor, (2) executive function, (3) language, (4) fluency—which is composed of three regions: BA9, BA44 and BA41 with overlap in both language and fluency domains, (5) sensory and (6) other. 0=no TDP-43 pathology identified, 1=mild TDP-43 pathology, 2=moderate TDP-43 pathology and 3=severe TDP-43 pathology. All cases were investigated for genetic diagnoses by either whole genome sequencing or repeat-prime PCR (for *C9orf72* status), and mutations are indicated in brackets after the case number.

ECAS, Edinburgh Cognitive and Behavioural Amytrophic lateral sclerosis Screen; TDP-43, 43 kDa Tar-DNA binding protein.

### Subdomain cognitive dysfunction detected by the ECAS relates to specific regional distribution of pathology

Three of the 27 patients demonstrated mild executive dysfunction when tested (one with pure executive dysfunction and two with a combined executive, language and fluency dysfunction) and all three had corresponding TDP-43 pathology at postmortem in the brain areas associated with executive functions (BA6, BA11, BA24, BA46 and BA9). Furthermore eight of the 27 patients demonstrated mild language impairment (BA44, BA41, BA20 and BA39) when tested with ECAS (six with pure language dysfunction and two with a combined executive, language and fluency dysfunction), all of whom had TDP-43 pathology in corresponding brain areas at postmortem. Four of the 27 patients demonstrated mild fluency dysfunction when tested with ECAS (two with pure fluency dysfunction and two with a combined executive, language and fluency dysfunction) and all four had corresponding TDP-43 pathology at postmortem in the brain areas associated with fluency (BA9, BA44 and BA41). There were no cases where cognitive impairment was present in the absence of TDP-43 pathology; however, there was a small subgroup (n=6) of patients who had TDP-43 pathology with no cognitive impairment (false negatives). These results taken together gave rise to the ECAS accurately predicting TDP-43 pathology with a positive predictive value of 100% and 100% specificity in executive function, language and fluency domains (figure 1B).

## Genetic status, cognitive impairment and pathology

Six out of the 27 cases identified had a genetic diagnosis of a *C9orf72* repeat expansion; all cases had extramotor TDP-43 pathology at postmortem, irrespective of cognitive impairment. This is in stark contrast to two patients in the cohort with a *SOD1* (*I114T*) mutation (Scottish founder mutation), who had no detectable TDP-43 pathology and no cognitive dysfunction (although one additional SOD1 individual was classified as ALSbi, but had no TDP-43 pathology), consistent with previous neuropathological and neuropsychological assessments.^9 25^ Of these six *C9orf72* repeat expansion cases, one had executive dysfunction and three had language impairment. We also identified two patients carrying a *NEK1* mutation, one of these patients had ALSbi with TDP-43 pathology and the other had no cognitive impairment and no evidence of extramotor TDP-43 pathology at postmortem.

### TDP-43 protein distribution is cell-type specific and differs between individuals

Analysis of the cell-type specific distribution of TDP-43 pathology was performed demonstrating three predominating patterns: (1) predominantly glial (22.2% of cases), (2) mixed glial and neuronal (59.3% of cases) and (3) predominantly neuronal TDP-43 pathology (7.4% of cases; figure 2). One-way ANOVA was performed to assess whether the cell-type specific distribution of TDP-43 pathology was associated with cognition in the regions assessed (figure 2D), demonstrating no significant difference (p>0.05; table 5). Indeed, there was no statistically significant difference between the groups for other demographic and clinical characteristics, including sex, anatomical onset of disease, median age at onset or death, disease duration or genetics (measured by one-way ANOVA with Bonferroni correction for multiple testing p>0.05; table 5). This is likely due to the small sample size evaluated and clearly warrants further investigation in a larger cohort or meta-analysis of existing data to evaluate conclusively whether these phenotypes impact on clinical characteristics of ALS and or FTD.

**Table 5:** Cell-type specific pathology in ALS

TDP-43 pathology	Glial	Mixed	Neuronal	SOD1/no TDP-43 pathology
Number of patients	6	16	2	3
Proportion of cohort	22.2	59.3	7.4	11.1
Sex (M/F)	4 M; 2 F	9 M; 7 F	1 M; 1 F	2 F; 1 M
Onset				
Limb	3	13	2	3
Bulbar	3	2	0	0
Both	0	1	0	0
Median age (range)				
At disease onset	56 (39–69)	56 (45–79)	60 (54–66)	45 (32–59)
At death	63 (43–70)	63 (50–84)	69 (62–76)	48 (40–64)
Median disease duration	58 (16–109)	43 (13–134)	108 (97–199)	67 (30–98)
Genetics				
No mutation	3	13	0	1
C9orf72	3	2	1	0
SOD1	0	0	0	2
NEK1	0	1	1	0
Impaired cognition (ECAS)				
Behavioural	0	2	0	1
Executive	0	0	1	0
Language	2	4	0	0
Fluency	1	1	0	0
Combined	0	2	0	0

Summary of clinical findings and demographics of cohort separated by cell-type specific TDP-43 pathology (as demonstrated in figure 2).

ALS, amyotrophic lateral sclerosis; ECAS, Edinburgh Cognitive and Behavioural ALS Screen; F, female; M, male; TDP-43, 43 kDa Tar-DNA binding protein.

## Discussion

We undertook an in-depth neuropathological analysis of 27 patients with non-demented ALS who had all undergone cognitive testing with the ECAS during life and demonstrated that all individuals with ALS-specific cognitive deficits (ALSci) had TDP-43 pathology in extramotor areas. Furthermore, a more detailed analysis of the types of cognitive impairment (executive/language/fluency) revealed a direct association between TDP-43 pathology and specific corresponding brain regions of the frontal and temporal lobes. Specifically we showed that (1) executive dysfunction was associated with TDP-43 pathology in the following regions: the orbitofrontal cortex (BA11/12), ventral anterior cingulate (BA24), dorsolateral prefrontal cortex (BA46 and BA9) and the medial prefrontal cortex (BA6); (2) language dysfunction was associated with TDP-43 pathology in the inferior frontal gyrus (Broca’s area; BA44/45), transverse temporal area (Heschl’s gyri; BA41/42), middle and inferior temporal gyri (BA20/21) and the angular gyrus (BA39); (3) fluency dysfunction was associated with TDP-43 pathology in the prefrontal cortex (BA9), inferior frontal gyrus (Broca’s area; BA44/45), ventral anterior cingulate (BA24) and the transverse temporal area (Heschl’s gyri; BA41/42) and (4) behavioural impairment was associated with TDP-43 pathology in the orbitofrontal cortex (BA11/12), ventral anterior cingulate (BA24) and medial prefrontal cortex (BA6). We recognise that a potential limitation of these data is the difference in time between ECAS testing during life and time of death. However, we demonstrate that there was no effect of time between ECAS and death on our data. As such we are confident that our conclusions from these data are robust and meaningful.

Staging of TDP-43 pathology at postmortem has been described previously and together with the current findings indicates that the pathological accumulation of TDP-43 is clearly related to the clinical manifestations of ALS and could be a promising biomarker.^9 26^ We chose to assess TDP-43 pathology using a semiquantitative scoring system to assess non-motor brain regions, rather than the scoring systems previously published by Brettschneider and colleagues which focused on the motor region and behavioural variant FTD.^9 27^ Using our system, we were able to assess abundance of TDP-43 pathology in specific brain regions associated with subdomains of the ECAS and also to identify cell-type specific patterns of TDP-43 pathology, allowing us to generate a specific data set relevant to cognitive testing. Here, we present the first evidence suggesting that ECAS could be used to accurately predict the presence of TDP-43 pathology in specific non-motor brain regions at post-mortem. Indeed, the ECAS is now used worldwide in ALS clinics (https://ecas.psy.ed.ac.uk), highlighting the potential impact and clinical utility of these findings. Cognitive changes are found in about half of cases of ALS and given that these clinical manifestations of the disease are likely related to TDP-43 accumulation, there is potential for targeted therapies to improve cognition aimed at reducing the burden of misfolded TDP-43. These findings raise the possibility of using ECAS as a stratification tool in clinical trials, aimed at reducing cognitive symptoms in at risk individuals. Given that no false positives would be identified, the use of the ECAS as a stratification tool would not result in anyone being incorrectly included in such a clinical trial.

Although there were some cases with mild involvement of BA19 (five cases) and hippocampal pathology (four cases), there was no associated functional (memory, visuospatial) impairment. We identified one case with language impairment that also had some mild BA19 pathology. In this case, there was no additional hippocampal pathology and it is therefore likely that they performed poorly on the story recall memory test due to other cognitive problems (eg, language) and not because a primary impairment in memory.

There were three predominating cell-type specific patterns of TDP-43 pathology: (1) predominantly glial (22.2% of cases); (2) mixed neuronal and glial (59.3% of cases) and (3) predominantly neuronal (7.4% of cases). This finding is clearly present in other published pathological data sets^28^ and images,^29^ however, has, to our knowledge, never been formally reported. While our data showed no statistically significant association with cognition in these brain regions, we do not have an adequate sample size to be able to comment more specifically on the implications of this finding on ALS pathogenesis. However, given that this cell-type specific distribution of TDP-43 pathology exists, it is crucial that animal and cell models accurately model these differing neuronal and glial pathologies in future experimental paradigms. There is a focus on neuronal models of ALS, which may not accurately model the 22.2% of cases that we found to have a predominantly glial pattern of TDP-43 pathology.

Furthermore, in line with previous neuropathological studies of patients with ALS, all *C9orf72* repeat expansion carrying patients and none of the patients with a *SOD1* mutation had TDP-43 pathology at postmortem and neither TDP-43 severity nor cell-type distribution was associated with disease progression. Our data therefore show that there are two predictors of TDP-43 pathology at postmortem: (1) cognitive dysfunction assessed by the ECAS and (2) *C9orf72*/*SOD1* status. Given the impact that cognition has on the quality of life of both patients and carers, future studies looking at the effects of therapies, targeting protein misfolding, on cognitive function in patients with ALS, independent of their effects on survival or motor function, are clearly warranted. Our data demonstrate the utility of the ECAS in predicting TDP-43 pathology in extra-motor brain regions in postmortem tissue. The primary advantage of a specificity of 100% is that no individual would be incorrectly included in a clinical trial aimed at reducing TDP-43 burden. However, the major limitation to this is the lower sensitivity of the ECAS in predicting this burden of pathology, meaning that some patients, who could plausibly benefit from such a trial, would not be identified. However, recently we identified a striking difference in spatial expression of a pathological marker called clusterin in mismatch cases that would not be identified by the ECAS.^24^ It is therefore plausible that biomarkers exist that could be developed to enable improved sensitivity to identify further individuals that may be missed by the ECAS testing. Crucially the ECAS may therefore have ever improving utility in (1) identifying patients for inclusion and (2) monitoring patients’ response to treatment, in clinical trials aimed at reducing pathological TDP-43 accumulation.
