# Supplementary material for: Executive, language and fluency dysfunction are markers of localised TDP-43 cerebral pathology in non-demented ALS
Source: J Neurol Neurosurg Psychiatry. 2019 Sep 12;91(2):149–57. doi: 10.1136/jnnp-2019-320807 (PMC6996101; doi:10.1136/jnnp-2019-320807)
Supplement: Supplementary data [file jnnp-2019-320807supp001.pdf]

Diagnosing ALS-Frontotemporal Spectrum Disorder using the ECAS
Abrahams 2018

ALS-FTSD has been defined in new diagnostic criteria by Strong et al. (2017). The following indicates how the ECAS can be used to aid the diagnostic process.

<u>For diagnosing ALSci</u>
A deficit in either ECAS Total OR ALS Specific Scores.

Justification: The recently revised diagnostic criteria for diagnosing ALS-frontotemporal spectrum disorder recommended that when ALS screening tests and brief assessments such as the ECAS are employed, published cut-offs are used to determine ALSci (Strong et al. 2017). Niven et al. (2015) demonstrated that maximum sensitivity and specificity to detect ALS specific cognitive impairment is achieved through combined use of a deficit in either the ALS-Specific or ECAS Total scores.

<u>For diagnosing ALSbi</u>
The presence apathy
OR the presence of two other behaviour symptoms as found in the ECAS behaviour interview (disinhibition, loss of sympathy, perseveration, change in eating behaviour, psychosis).

Loss of insight is not specifically measured by the ECAS but can be included through clinical judgement as one of the other behaviour symptoms

<u>For diagnosing ALS-FTD</u>
Evidence of progressive deterioration in behaviour and/or cognition by observation or history
And
The presence of at least 2 behaviour symptoms (disinhibition, apathy, loss of sympathy, perseveration, eating behaviour) in addition to a deficit in ALS-Specific ECAS Score,
OR the presence of at least 2 behaviour symptom together with psychotic symptoms and/or loss of insight.

Diagnosis should be made by an experienced clinician or discussed within the MDT. It should be noted that in accord with the diagnosis of behavioural variant FTD diagnosis can be made without the presence of cognitive impairment.

Strong et al 2017 have an additional criteria which is the presence of language impairment meeting criteria for Semantic Dementia or Non-Fluent progressive aphasia (e.g. Gorno-Tempini et al. 2011). These conditions are rare (Saxon et al. 2018), diagnosis is complex particularly if the patient has bulbar dysfunction and should be undertaken by a neuropsychologist. The ECAS can be used to aid diagnosis together with other neuropsychological tests.
